# The transcription factor SoxD controls neuronal guidance in the *Drosophila* visual system

**DOI:** 10.1038/s41598-018-31654-5

**Published:** 2018-09-06

**Authors:** Esteban G. Contreras, Tomás Palominos, Álvaro Glavic, Andrea H. Brand, Jimena Sierralta, Carlos Oliva

**Affiliations:** 1 Department of Neuroscience and Biomedical Neuroscience Institute, Faculty of Medicine, Universidad de Chile, Independencia, 1027 Santiago, Chile; 2 Center for Genome Regulation, Faculty of Sciences, Universidad de Chile, Las Palmeras, 3425 Nuñoa Santiago, Chile; 3 Department of Cellular and Molecular Biology, Faculty of Biological Sciences, Pontificia Universidad Católica de Chile, Av Libertador Bernardo O’Higgins 340, Santiago, Chile; 4 The Gurdon Institute and Department of Physiology, Development and Neuroscience, University of Cambridge, Tennis Court Road, Cambridge, CB2 1QN United Kingdom

## Abstract

Precise control of neurite guidance during development is essential to ensure proper formation of neuronal networks and correct function of the central nervous system (CNS). How neuronal projections find their targets to generate appropriate synapses is not entirely understood. Although transcription factors are key molecules during neurogenesis, we do not know their entire function during the formation of networks in the CNS. Here, we used the *Drosophila melanogaster* optic lobe as a model for understanding neurite guidance during development. We assessed the function of Sox102F/SoxD, the unique *Drosophila* orthologue of the vertebrate SoxD family of transcription factors. SoxD is expressed in immature and mature neurons in the larval and adult lobula plate ganglia (one of the optic lobe neuropils), but is absent from glial cells, neural stem cells and progenitors of the lobula plate. SoxD RNAi knockdown in all neurons results in a reduction of the lobula plate neuropil, without affecting neuronal fate. This morphological defect is associated with an impaired optomotor response of adult flies. Moreover, knocking down SoxD only in T4/T5 neuronal types, which control motion vision, affects proper neurite guidance into the medulla and lobula. Our findings suggest that SoxD regulates neurite guidance, without affecting neuronal fate.

## Introduction

The proper formation of synaptic connections is essential for nervous system function in a mature organism. A central step during this process is the navigation of developing axons and dendrites during nervous system development. In this stepwise process, growth cones are presented with guidance cues at different choice points allowing neurites to reach their correct targets. Regulation of the expression pattern of guidance receptors and cues is fundamental for correct neuronal wiring, and several transcription factors play a main role in this process^1,2^.

**Figure 1:**
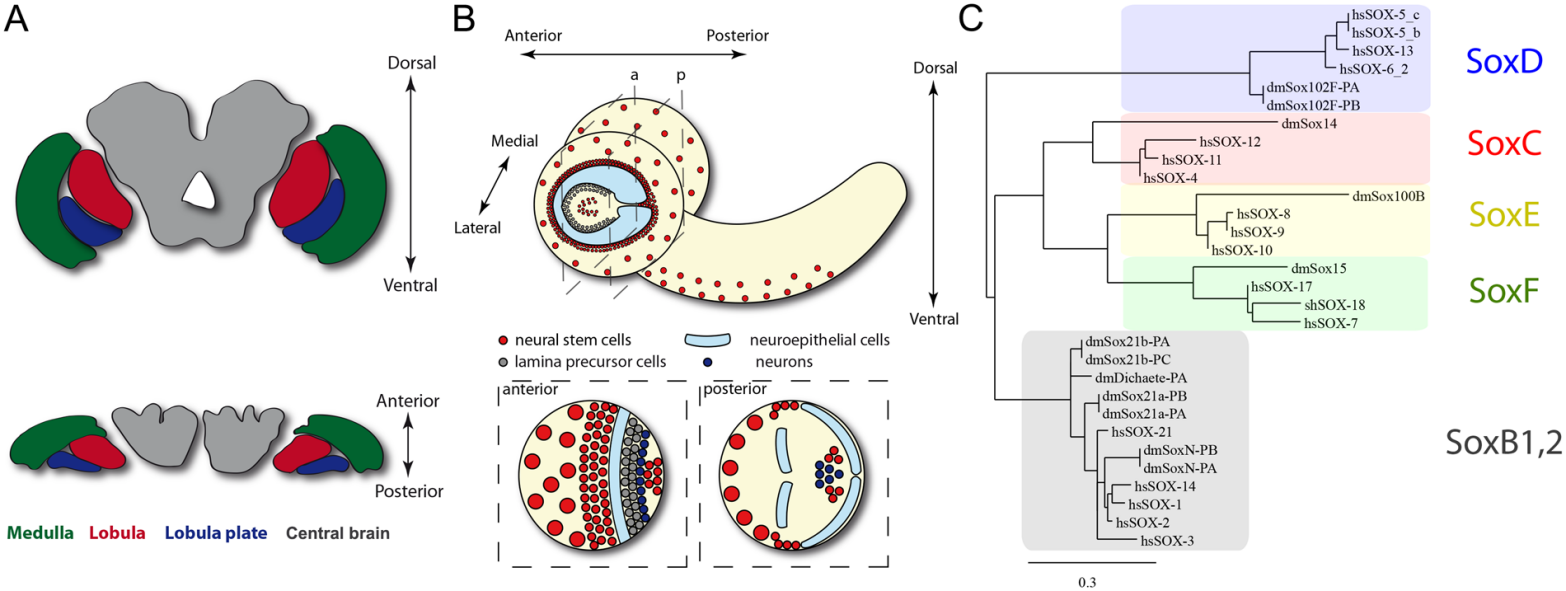
The Drosophila brain and the Sox family of transcription factors. (**A**) Schematic representation of the *Drosophila melanogaster* adult brain. The optic lobe medulla (green), lobula (red) and lobula plate (blue) neuropils are represented. The neuronal cell bodies are located in the periphery of the brain and not in the neuropils. (**B**) Diagram of the larval brain showing and anterior and posterior sections of a brain lobe. Neuroepithelial cells are shown in cyan, neural stem cells (neuroblasts) in red, lamina precursor cells (LPCs) in gray and neurons in blue. (**C**) Phylogenetic analysis of the human and *Drosophila* Sox family of transcription factors. The two isoforms of Sox102F/SoxD cluster with human SoxD members: SOX-5, SOX-6 and SOX-13.

The *Drosophila melanogaster* visual system is composed of the retina and the optic lobe, which is divided into four ganglia: lamina, medulla, lobula and lobula plate (see Fig. 1A). The visual inputs travel from the retinal photoreceptors through different optic lobe neurons, where this information is processed, triggering behavioural responses. Correct connectivity between optic lobe neurons is fundamental for sensing visual information^3–5^. In the past few years, several studies have characterised how transcription factors regulate the development and neuronal composition of the optic lobe. However, while the development of the lamina and medulla has been extensively studied^6–8^, research has only recently been focused on the development of the lobula complex (lobula and lobula plate)^8,9^.

The Sox (Sry Box) family of transcription factors is a key regulator of embryonic development^10^. These transcription factors bear a conserved DNA binding domain known as the SRY-related High Mobility Group-box (HMG-box), which was first described in the Sry protein that is fundamental for sex determination in mammals^11^. The Sox family of proteins is subdivided into nine groups, depending on the amino acid composition of their HMG-box^10,12^. Vertebrate genomes encode approximately 20 members of the Sox family, whereas only eight members have been described in the fruit fly^13^. The Sox transcription factors do not activate or repress gene expression themselves, but act together with partner factors that determine the modulation of target genes^12^.

Sox proteins are important during neural development and different groups of Sox transcription factors are responsible for similar neurodevelopmental processes across species. For instance, SoxB1 group members work in early neurogenesis in vertebrates and invertebrates. The vertebrate SoxB1 protein, Sox2, participates in early events of central and peripheral nervous system development^14^. In a similar manner, the SoxB1 orthologues in *Drosophila*, SoxNeuro (SoxN) and Dichaete, are also required for proper neurogenesis^15,16^ and the formation of neural stem cells during development^17^. On the other hand, SoxD proteins are generally involved later during nervous system development. Vertebrate Sox5 and Sox6 regulate neural stem cell proliferation, neuronal diversity, neuronal migration and projection formation^12,18–20^. Similarly, the *Drosophila* SoxD orthologue is necessary for the development of the nervous system and loss of SoxD function affects synaptic bouton development at the neuromuscular junction and dendritic arborisation in sensory neurons^21,22^.

Here we analyse the role of the *Drosophila melanogaster* orthologue of the SoxD family: Sox102F/SoxD during optic lobe development. We show that SoxD is expressed in all optic lobe ganglia and is involved in the morphogenesis of the lobula plate neuropil. RNAi-mediated SoxD knockdown in developing neurons severely alters the morphology of the lobula plate ganglia. These morphological defects are not a consequence of changes in the fate of lobula plate neurons, but result from an alteration in the normal pattern of axon and dendrite formation. Associated with the defects in lobula plate morphology, the fly optomotor response is also impaired upon SoxD downregulation in lobula plate neurons. These results are consistent with the observation that Sox5 is involved in neuronal migration and axon pathfinding in mice, denoting that SoxD function is evolutionary conserved.

## Results

### ***soxD*** mRNA is localised in the developing optic lobe

With the goal of finding new genes required for *Drosophila* optic lobe development, we assessed a group of candidate genes that are enriched in the optic lobe^23^. Among these, we decided to characterise CG11153/Sox102F and its role during nervous system development. A previous study demonstrated that Sox102F is expressed in the larval optic lobe^13^, suggesting a function in the development of this region of the brain.

Sox102F has been described as the *Drosophila* homolog of the SoxD family^13,22,24^. Both Sox102F protein products (Sox102F-PA and Sox102F-PB) cluster with *Homo sapiens* SoxD family proteins in a phylogenetic analysis (see blue cluster in Fig. 1C). Sox102F exhibits high homology to all SoxD family members: 77% identity with hsSox6, 74% with hsSox5 and 72% with hsSox13. Previously, Sox102F was considered the *Drosophila melanogaster* homolog of vertebrate Sox5^21,25^; however, the high level of identity to all members of the SoxD group (Sox6, Sox5 and Sox13) suggests that Sox102F is the ortholog of the entire family. Therefore, we decided to rename CG11153/Sox102F as SoxD.

**Figure 2:**
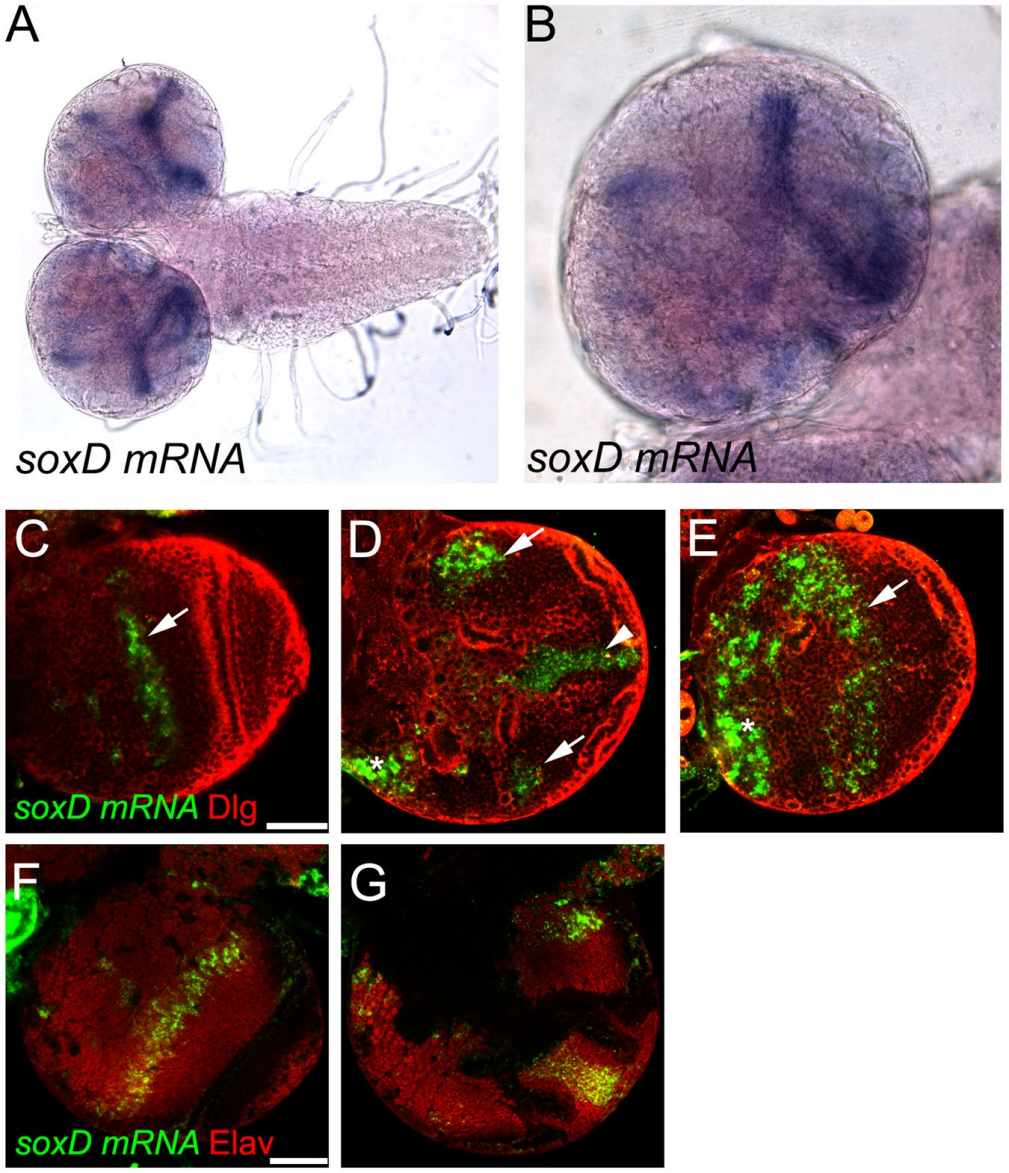
*soxD* is expressed in the larval optic lobe. (**A**,**B**) Larval brain *in situ* hybridisation using a specific probe against *soxD*. (**C–E**) Fluorescent *in situ* hybridisation of larval brain for *soxD* (green) and co-stained with antibody against Discs large (Dlg, red), different sections in the antero-posterior axis. (**F**,**G**) Fluorescent *in situ* hybridisation of larval brain for *soxD* (green) and co-stained with anti-Elav (red), different sections in the antero-posterior axis. Arrows show signal in the medulla, while arrowheads show signal in the lobula complex. Scale bars are 50 µm.

In order to analyse *soxD* expression pattern during optic lobe development we generated an RNA probe against all *soxD* mRNA isoforms. Whole mount *in situ* hybridisation showed high levels of *soxD* expression in the larval optic lobe (Fig. 2A,B, see Fig. 1B for a scheme of the optic lobe) as previously reported^13^. Due to the complex morphology of the larval optic lobe, we performed fluorescent *in situ* hybridisation (FISH) to obtain higher resolution in order to distinguish the cell types that express *soxD*. We observed that *soxD* mRNA was highly expressed in the optic lobe medulla and lobula complex (see arrow and arrowheads in Fig. 2C–E). Additionally, the *soxD* mRNA FISH signal was also present in the developing central brain (see star in Fig. 2D,E).

The developing larval brain is composed of neural cells at different levels of differentiation, including neural stem cells, intermediate progenitors (INPs and GMCs), developing neurons and glial cells. To assess in which cell type *soxD* mRNA was present, we co-stained *soxD* FISH with the pan-neuronal marker Elav, which is expressed early during neurogenesis after neuronal commitment. *soxD* FISH staining was observed in Elav-positive cells both in the optic lobe and the central brain (Fig. 2F,G). Therefore, *soxD* is expressed in developing neurons but not in neural stem cells or glia.

### Medulla neurons, lobula plate neurons and lamina progenitors express SoxD protein

**Figure 3:**
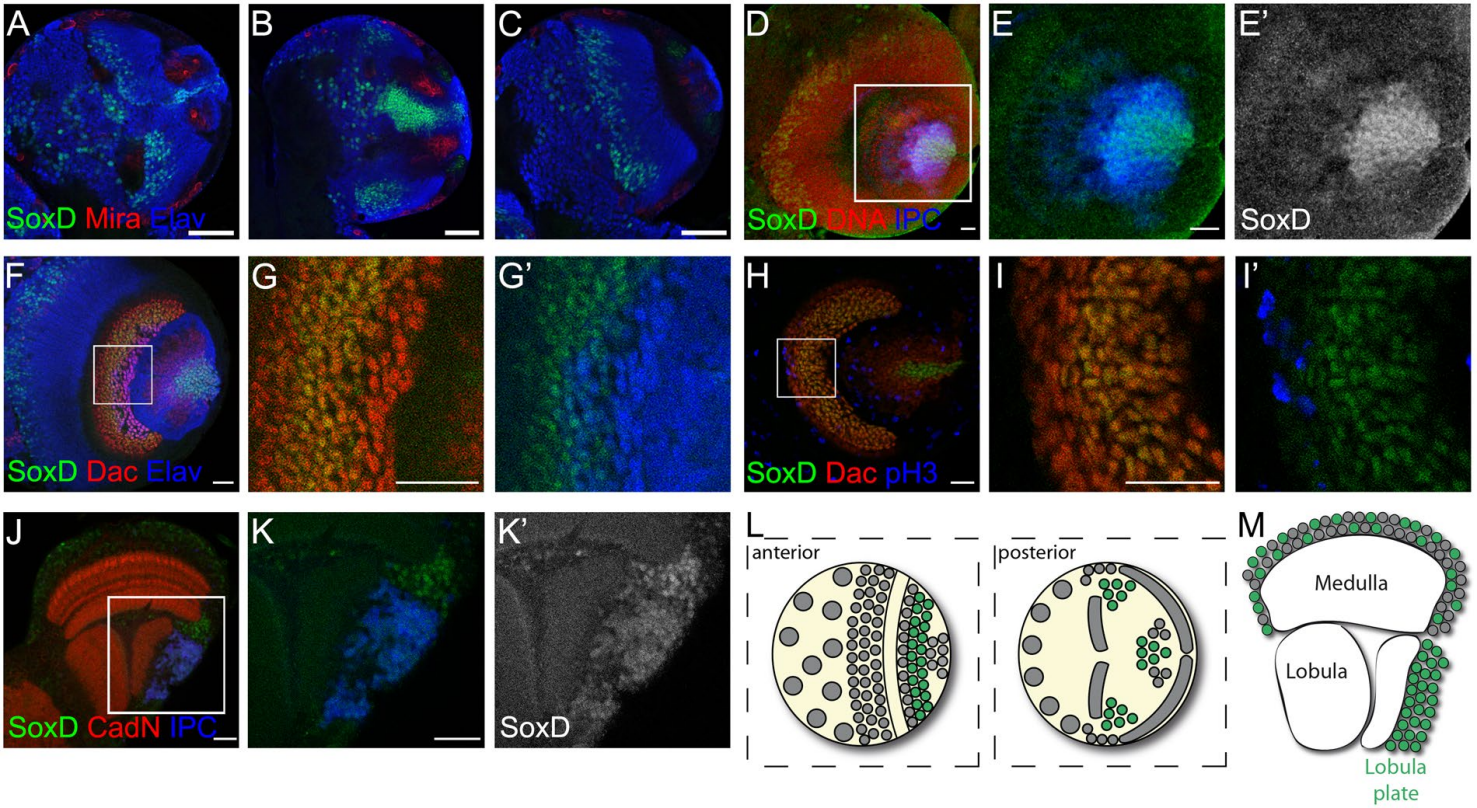
SoxD protein is found in medulla neurons, lobula complex neurons and lamina progenitors. (**A**–**C**) Immunofluorescence of Sox:GFP (green) larval brains stained for Mira (red) and Elav (blue), different sections in the antero-posterior axis are shown. (**D**–**G**) Staining of larval brains expressing IPC-LexA, LexAop-mCherry (blue), SoxD:GFP (green) and DNA (red), lateral view. (**F–I’**) Staining of SoxD:GFP (green) larval brains for Dac (red) and (**F,G’**) Elav (blue) or (**H,I’**) pH3 (blue), lateral view. (**J,K’**) Immunofluorescence of an adult optic lobe of IPC-LexA, LexAop-mCherry (blue), SoxD:GFP (green) genotype and co-stained for CadN (red), lateral view. Scale bars are (**A–C**) 50 µm and (**D–K**’) 20 µm. (**L,M**) Schematic representation of the (**L**) larval and (**M**) adult optic lobe showing expression of SoxD in green, horizontal view.

Owing to the lack of an antibody against *Drosophila* SoxD, we used a *Drosophila* line in which SoxD is tagged with GFP at its endogenous locus (SoxD:GFP). We observed that the SoxD:GFP expression pattern resembled *soxD* FISH in the larval brain (compare Fig. 2C,D with Fig. 3A–C) and SoxD:GFP co-localised with Elav staining in nuclei (Fig. 3A–C). Interestingly, SoxD:GFP was expressed only in subpopulations of medulla and lobula complex developing neurons; many Elav-positive neurons did not show SoxD:GFP expression. To determine whether SoxD was expressed in neural stem cells (neuroblasts), we stained for the neuroblast marker Miranda (Mira)^26,27^ observing that the SoxD:GFP signal was not present in Mira-positive neuroblasts in the larval brain (Fig. 3A–C). Furthermore, we checked whether SoxD was expressed in glial cells using the pan-glial marker Reverse polarity (Repo), finding that SoxD:GFP signal was not detected in Repo-positive glial cells (see Supplementary Figure S1). These results confirm that SoxD is expressed in developing neurons of the medulla and lobula complex, but not in neural stem cells or progenitors of these ganglia.

The developing lobula complex generates the adult lobula and lobula plate neurons, however, these ganglia cannot be differentiated by their morphology during larval development. To overcome this limitation, we used the LexAop-mCherry fluorescent reporter in combination with the IPC-lexA line, which drives expression in the lobula plate lineage^28^, to identify this subpopulation. SoxD:GFP signal was observed in a population of developing IPC-positive neurons in the larval brain (Fig. 3D,E’), suggesting that SoxD is involved in lobula plate development. Interestingly, in the adult optic lobe weak levels of SoxD were present in a sparse group of neurons in the medulla and most lobula plate neurons (Fig. 3J,K’).

Analysing the SoxD:GFP expression pattern, we noted that it was also present in the developing lamina of the larval optic lobe. By co-staining with the lamina marker Dachshund (Dac) we observed that SoxD is expressed in a stripe of Dac-positive cells (Fig. 3F–I). Interestingly, most SoxD-positive cells had no or very low levels of Elav staining (see Fig. 3F,G’), suggesting that the SoxD-positive cells are lamina precursor cells (LPCs). LPCs are progenitors that divide once and terminally differentiate into neurons^29^. We stained larval brains with the mitotic marker phospho-Histone H3 (pH3) to identify LPCs during mitosis, finding that mitotic LPCs did not express SoxD (Fig. 3H,I’). These results suggest that SoxD is transiently expressed after LPC mitosis and that its levels are rapidly downregulated at the onset of neuronal differentiation.

In summary, SoxD is expressed in neurons of the medulla and lobula plate, and in the lamina precursor cell progenies before neuronal fate commitment (Fig. 3L), whereas in the adult optic lobe, low levels of SoxD are found in most of the lobula plate and in a sparse group of medulla neurons (Fig. 3M).

### SoxD is necessary for proper lobula plate development

**Figure 4:**
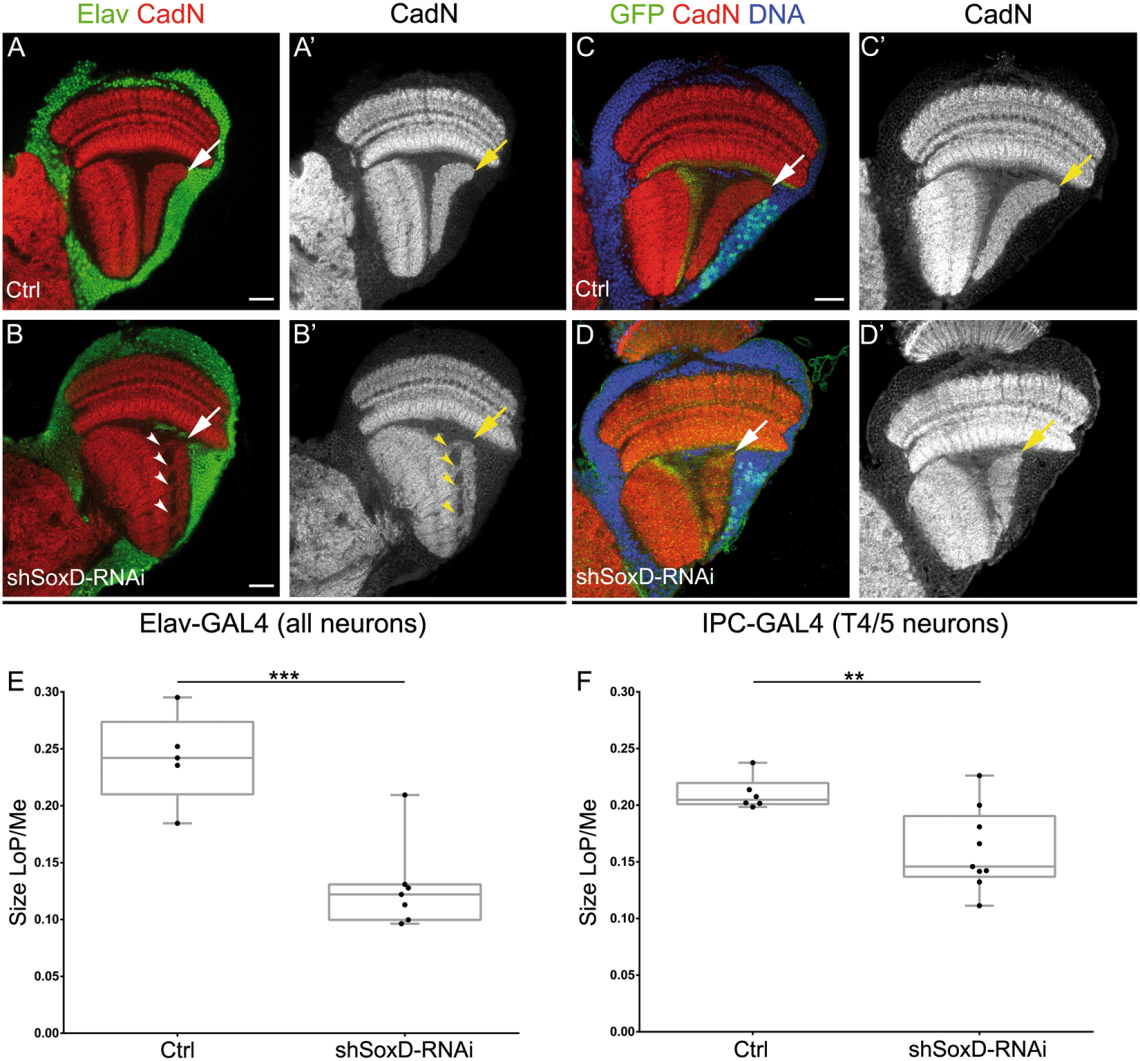
Lobula plate development requires SoxD function. (**A,B’**) Immunostaining of (**A,A’**) control and (**B,B**’) shSoxD-RNAi knockdown adult optic lobes against Elav (green) and CadN (red or gray). Knockdown was performed in all neurons during development using the Elav-GAL4 driver. (**C,C’**) Immunostaining of control and (**D,D’**) shSoxD-RNAi knockdown adult optic lobes using the IPC-Gal4 driver. Arrows indicate the lobula plate neuropil and arrowheads show bridges between the lobula and lobula plate neuropils. Scale bars are 20 µm. (**E**,**F**) Graphs showing the quantifications of the lobula plate (LoP) size normalised to the size of the medulla (Me) of control and shSoxD-RNAi knockdown in (**E**) all neurons and (**F**) T4/T5 neurons. Student’s t-test was performed. ** and *** are p-value < 0.01 and 0.001 respectively.

To understand SoxD function during the lobula plate development, we took advantage of available transgenic RNAi lines to knockdown SoxD using the GAL4/UAS system^30^. We targeted SoxD-RNAi to all developing neurons using the Elav-GAL4 driver, obtaining viable and fertile adults. We stained adult brains for the pan-neuronal marker Elav and the neuropil marker Cadherin-N (CadN). Interestingly, we observed a significant reduction in the size of the lobula plate neuropil after SoxD knockdown (see arrows in Fig. 4A,B’ and E). Furthermore, although in wild-type animals lobula and lobula plate neuropils are clearly segregated, upon SoxD knocking down, ectopic bridges are formed between these neuropils (see arrowheads in Fig. 4B,B’). Importantly, these phenotypes were observed using three different RNAi lines, indicating that they are indeed specific of SoxD knockdown.

Surprisingly, no apparent changes in the morphology of the adult lobula plate cortex were observed after knocking down SoxD (Fig. 4B). Although SoxD RNAi was expressed in all developing neurons, medulla morphology was not affected (Fig. 4B,B’). Similarly, when SoxD was knockdown in the developing lamina, using upd-GAL4^E132^ driver^31^, we did not observe any defects in the adult lamina morphology (Supplementary Figure S2). These results suggest that, although SoxD is expressed in some medulla and lamina cells, its function is not required for the development of these neuropils at least at the morphological level.

To confirm the SoxD knockdown phenotype, we used a genetic approach. Due to the lack of a proper *soxD* null allele, we used a viable hypomorphic allele in combination with a chromosome deletion. We crossed *soxD*^*MI01054*^ allele, a MiMIC transposon^32^ insertion that eliminates one isoform of *soxD*, with a deficiency chromosome that deletes several loci including the entire *soxD* locus (*Df(4)O2*, Supplementary Figure S3A). Thus, we observed that the lobula plate neuropil was less defined and formed bridges with the lobula neuropil (see Supplementary Figure S3B-C’), resembling the phenotype generated by SoxD-RNAi (Fig. 4B,B’). However, the size of the lobula plate was not affected in the *soxD* mutant animals compared to the hemizygous controls (check Supplementary Figure S3D).

To narrow down the expression of the SoxD-RNAi only to developing lobula plate neurons, we used the previously described driver IPC-GAL4^33^. Similar to the results obtained with Elav-GAL4, we observed a significant reduction in the size of the lobula plate neuropil compared to control animals (Fig. 4F) but not a change in the size of the cortex where the neuronal somas reside (Fig. 4C,D’).

The reduction in the size of the lobula plate neuropil observed after SoxD knockdown could be due to a defect during development or to neurodegeneration of adult neurons. To discriminate between these two possibilities, we knocked down SoxD using insc-GAL4, a driver that is only expressed early during neurogenesis (in neuroblasts and ganglion mother cells, while GAL4 levels persist in developing neurons), but not in fully differentiated neurons. Thus, we analysed the morphology of the lobula plate during pupal development observing a reduction in the its neuropil at 48 and 72 hrs after puparium formation (APF) and in adult animals that expressed SoxD-RNAi (see Supplementary Figure S4). Since the defects were observed as early as 48 hours APF when only early neurogenesis was targeted, SoxD seems to be required for the development of the lobula plate neuropil. To determine whether the phenotype observed in the adult lobula plate after SoxD knockdown could be generated by defects in neuronal fate commitment, we used two markers of lobula plate T4/T5 neuronal fate: Acj6 and Dac^33,34^. When the SoxD-RNAi was expressed in T4/T5 lobula plate neurons, using IPC-GAL4, neither Acj6 nor Dac expression was affected (see Supplementary Figure S5A–D’), suggesting that T4/T5 fate is not determined by SoxD. To confirm the efficiency of this knockdown, we checked SoxD:GFP levels when SoxD-RNAi was expressed using IPC-GAL4. We observed a significant reduction in SoxD:GFP only in the lobula plate, while the expression in the medulla was not affected (Supplementary Figure S5E,F’). Together these results indicate that SoxD function is necessary for proper formation of the lobula plate neuropil during development without affecting neuronal fate.

### SoxD is required for the adult optomotor response

**Figure 5:**
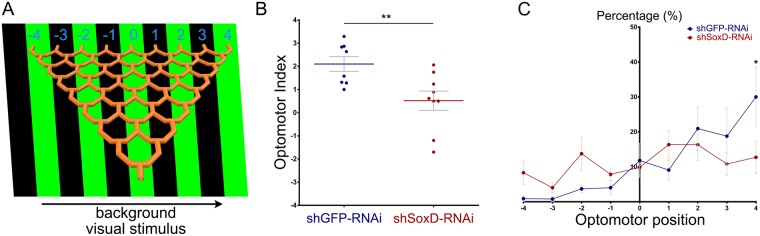
SoxD knockdown affects optomotor response. (**A**) Diagram of the optomotor paradigm. An eight-choice maze is attached to a screen that displays green and black strips moving to the right. Flies enter the maze from its bottom and exit in different optomotor positions in the top of the maze. (**B**) Graph showing the Optomotor Index (OI) of control shGFP-RNAi and shSoxD-RNAi driven by IPC-GAL4. (**C**) Plot showing the percentage of flies in each optomotor position of control shGFP-RNAi (blue lines) and shSoxD-RNAi (red lines) driven by IPC-GAL4. Mann-Whitney test was used for OI, while 2-way ANOVA with Bonferroni’s multiple comparison test were used for optomotor positions.* and ** are p-value < 0.05 and 0.01 respectively.

Given the role of the lobula plate in the processing of the motion vision in insects, we assessed whether the morphological defects resulting from the reduction in SoxD function could affect the animal visual response. For this we took advantage of an established paradigm to evaluate visual motion detection response, in which a population of flies walk through an eight-point choice maze with a moving visual stimulus (green stripes moving to the right, see Fig. 5A)^35,36^. At each choice point of the maze, the flies turn left or right, according to the stimulus direction, to end in one of nine collection tubes at the other end of the maze. Depending on the collection tube, each fly is assigned an optomotor position value (from -4 to 4) and an Optomotor Index (OI) is calculated for the group.

Adult control flies expressing an RNAi against GFP under the control of the IPC-GAL4 driver showed an average OI of 2.1, which reflected the trend of flies to turn right in response to the moving visual stimulus (Fig. 5B). However, when SoxD was knocked down the average OI was significantly reduced to 0.5, indicating a lack of optomotor response (Fig. 5B). This could also be observed when the percentage of flies in each optomotor position was plotted for both groups. Hence, control flies had a marked trend to end in positive optomotor positions, whereas SoxD-RNAi flies showed no preference (Fig. 5C). Together these results strongly suggest that SoxD is necessary for correct neuronal wiring in the lobula plate, which is necessary for controlling optomotor behaviour.

### SoxD controls neurite targeting of T4/T5 neurons

**Figure 6:**
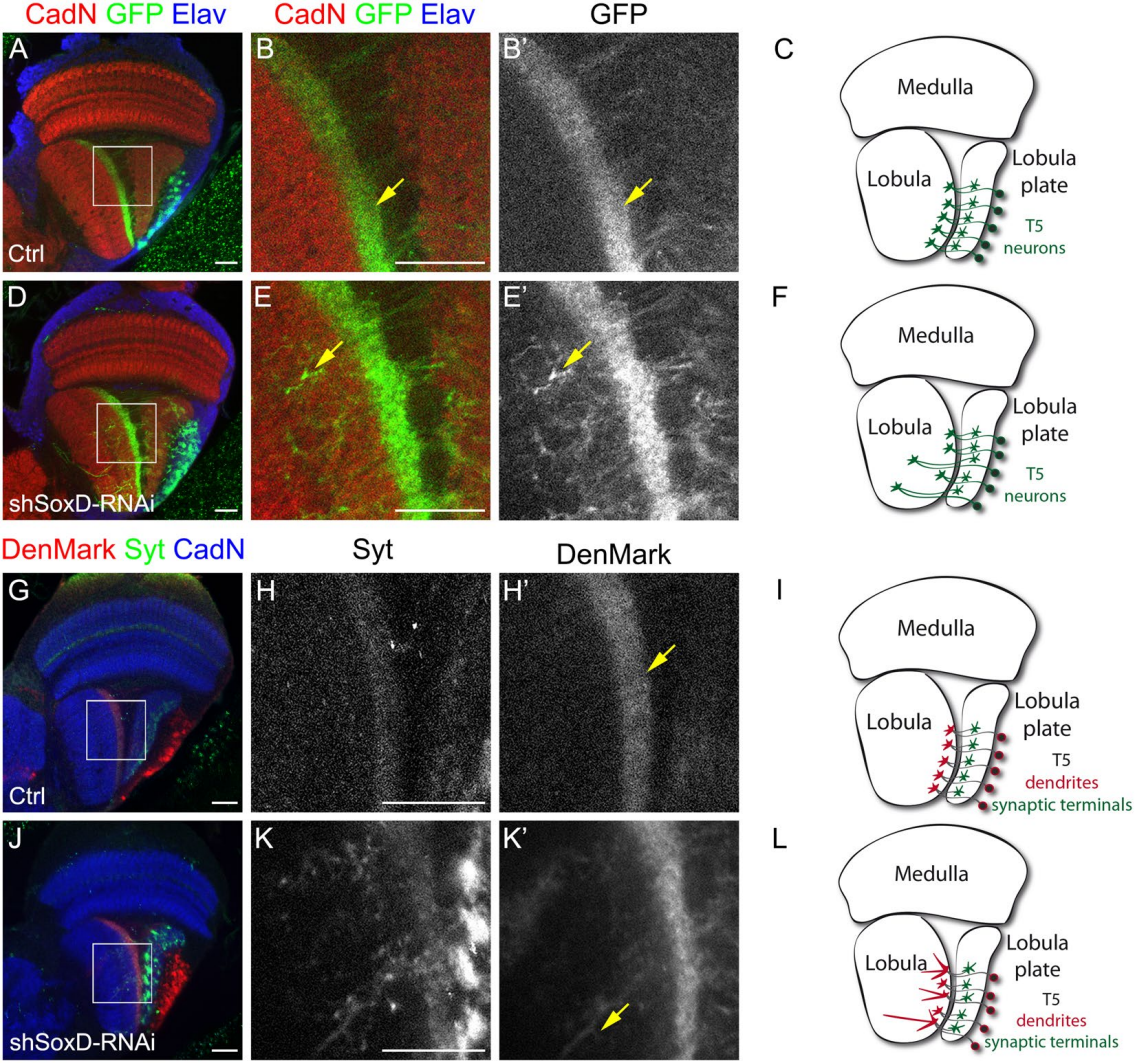
SoxD controls T5 arborisation in a neuronal-autonomous manner. (**A,B’**, **D,E**’) Immunofluorescence of adult optic lobes of R42H07-GAL4, UAS-mCD8-GFP (**A,B**’) control and (**D,E**’) shSoxD-RNAi knockdown, against GFP (green), Elav (blue) and CadN (red). (**C**,**F**) Schemes showing lobula plate T5 neurons in (**C**) control and (**F**) SoxD knockdown backgrounds. (**G,H**’, **J,K**’) Immunostaining of adult optic lobes of R42H07-GAL4, UAS-DenMark, UAS-syt-eGFP (**G,H’**) control and (**J,K’**) shSoxD-RNAi knockdown, against GFP (green), RFP (red) and CadN (blue). **(I,L)** Schemes showing lobula plate T5 neurons in (**I**) control and (**L**) SoxD knockdown backgrounds. Arrows point to (**B**,**B’**, **E**,**E’**) neurites or (**H,H’, K,K’**) dendrites in location. Scale bars are 20 µm.

To explore the phenotype observed after SoxD knockdown in more detail, we directed the expression of SoxD-RNAi to a specific population of lobula plate neurons. For this, we used R42H07-GAL4^37,38^ to drive the expression of SoxD-RNAi and a membrane-tagged GFP specifically in T5 neurons of the lobula plate, part of the motion detection processing circuit that controls optomotor behaviour^38^. T5 are unipolar neurons that extend a single projection to the Lo1 layer of the lobula neuropil where dendrites are formed (arrows in Fig. 6B,B’). The projection then goes back to the lobula plate neuropil where synapses are formed (Fig. 6A–C)^39^. After SoxD knockdown in T5 neurons, although strong GFP signal was observed in the Lo1 layer, several projections were mistargeted into deep layers of the lobula neuropil (see Fig. 6D–F). Interestingly, SoxD-RNAi cell bodies localised in the lobula plate cortex in a similar fashion to control brains, with no indication of a fate change into lobula neurons.

In order to test whether SoxD knockdown can affect neurite arborisation of another neuronal type in the lobula plate, we checked T4 neurons. Similar to T5 neurons, T4 neurons also form part of the motion detection circuit in the lobula plate^38^. We targeted the SoxD-RNAi to T4 and T5 neurons using IPC-GAL4 in combination with R42F06-GAL4^37,38^, which is strongly expressed in adult T4/T5 neurons, and membrane-tagged tdTomato. We observed that T4 neurites in control conditions reached the innermost layer of the medulla^39^, however, after SoxD knockdown T4 neurites continued projecting to outer medulla layers (see arrows in Supplementary Figure S6). This neurite guidance phenotype was also observed in T5 neurons using this driver combination (Supplementary Figure S6D,D’), suggesting that SoxD is necessary for, at least, T4 and T5 neurite targeting.

Due to the unipolar characteristic of T4 and T5 neurons, we could not distinguish whether dendrites or axons failed to properly project into other lobula layers after SoxD knockdown. To address this question, we expressed a dendrite marker (DenMark^40^) and a synaptic terminal fluorescent marker (Synaptotagmin 1, syt-eGFP) in T5 neurons. In control brains T5 dendrites in the lobula were restricted to a Lo1 layer, while synaptic terminals were found in the outer layers of the lobula plate (Fig. 6G–I). However, when SoxD was knocked down in T5 neurons, dendritic projections were found in deep layers of the lobula (Fig. 6J–L). In the case of the synaptic terminals, syt-eGFP was distributed in the entire lobula plate, lacking the organised pattern observed in control brains (compare Fig. 6G with Fig. 6J). Furthermore, syt-eGFP was also observed in the lobula layers together with the dendritic marker (see Fig. 6K). These results strongly support that SoxD controls proper terminal differentiation of lobula plate neurons, regulating neurite guidance.

### SoxD is sufficient to impair neurite guidance

**Figure 7:**
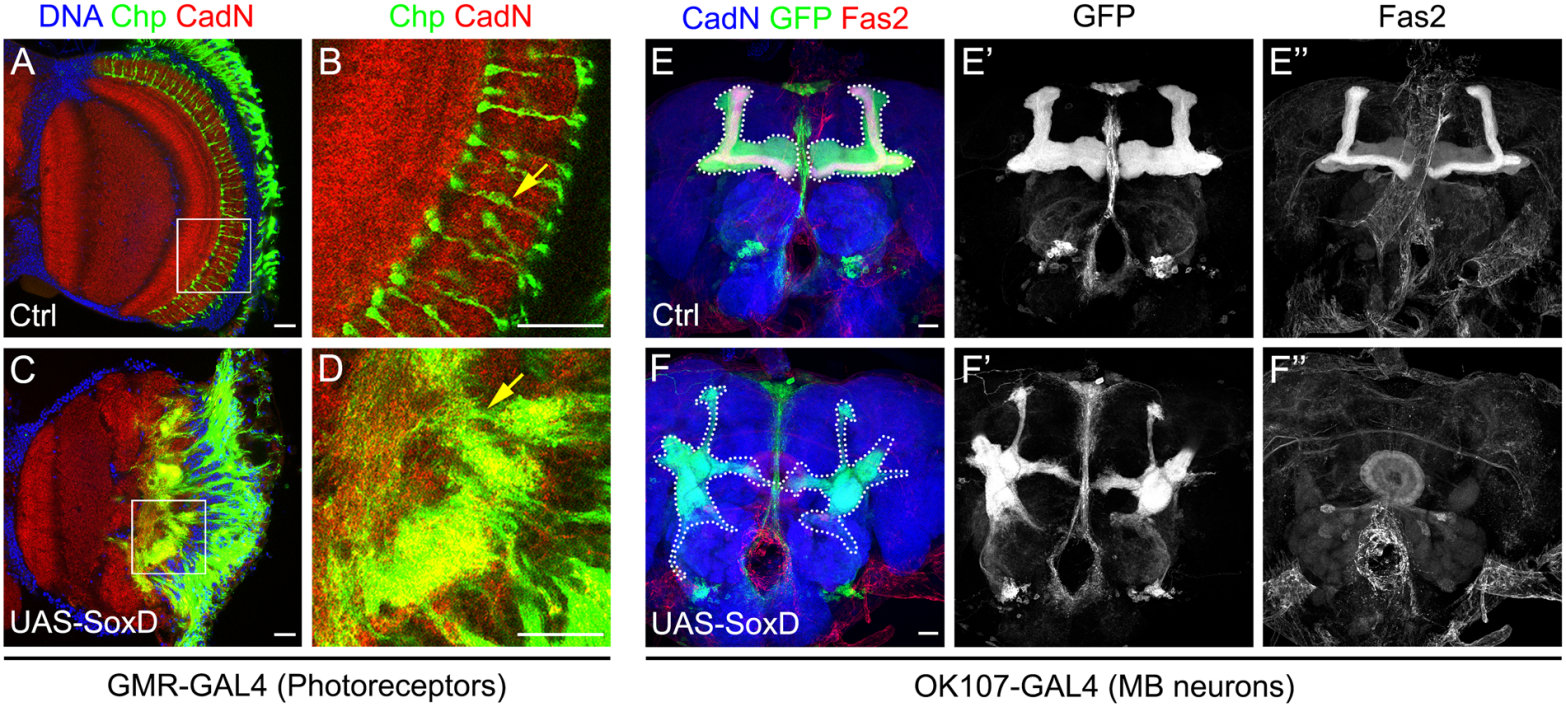
SoxD misexpression disrupts neurite guidance. (**A–D**) Immunofluorescence of adult optic lobes of GMR-GAL4 (**A**,**B**) control and (**C**,**D**) UAS-SoxD, stained for Chp (green), CadN (red) and DNA (blue). Arrows point to photoreceptors in the medulla. (**E,F**’) Immunofluorescence of adult central brain of OK107-GAL4, UAS-mCD8-GFP (**E,E”**) control and (**F,F”**) UAS-SoxD, stained for GFP (green or gray), Fas2 (red or gray) and CadN (blue). The structure of the mushroom body is highlighted by dotted lines. Scale bars are 20 µm.

As SoxD is necessary for neurite guidance in the lobula plate, we wonder whether ectopic expression of SoxD could affect neurite targeting in other contexts. SoxD is not expressed in photoreceptors during development (Supplementary Figure S7A,A”), therefore, we misexpressed SoxD (UAS-SoxD) using GMR-GAL4 and analysed adult optic lobes stained for the photoreceptor marker Chaoptin (Chp). In control optic lobes, R7 and R8 photoreceptors reached the M6 and M3 medulla layers respectively (Fig. 7A,B). However, after SoxD misexpression, we observed a massive disruption in photoreceptor targeting that compromised the morphology of the medulla neuropil. Thus, photoreceptor axonal development was severely affected, while the differentiation marker Chp maintains its expression (Fig. 7C,D).

To confirm that SoxD is able to affect neurite targeting, we misexpressed SoxD in mushroom body neurons that do not express SoxD during development (Supplementary Figure S7B,B”). To analyse the mushroom bodies, we expressed a membrane-bounded GFP using OK107-GAL4 driver. and stained for the *α*/*β* and γ Kenyon cell marker Fasciclin 2 (Fas2)^41^. When SoxD was misexpressed in mushroom body neurons, the Kenyon cell lobes were disrupted and neuronal projections were aberrantly targeted to different regions of the central brain (Fig. 7E,F’). Interestingly, Fas2 expression was strongly diminished after SoxD misexpression (compare Fig. 7E” and F”) suggesting that mushroom body neuronal fate was also altered by SoxD ectopic expression. Finally, we overexpressed SoxD in T5 neurons using the R42H07-GAL4, observing some mistargeted neurites in deeper lobula layers (Supplementary Figure S7C–F’). Together these experiments support the conclusion that SoxD is sufficient for affecting neurite guidance.

## Discussion

Transcriptional control is a key regulator of many cellular processes including neuronal differentiation and axon/dendrite guidance. A large number of transcription factors have been identified to play a role during neurite guidance. Here we describe the function of Sox102F, the only member of the SoxD family in *Drosophila melanogaster*, in the regulation of neurite targeting in the visual system. According to our phylogenetic analysis, Sox102F has high homology to human Sox5, Sox6 and Sox13 transcription factors; therefore, we propose to rename this protein SoxD. During larval brain development, SoxD is expressed in neurons of the medulla and the lobula complex, while in the lamina, SoxD is transiently expressed before LPC differentiation into neurons. SoxD knockdown in all neurons or in lobula plate neurons severely affects the morphology of the lobula plate neuropil, impairing fly optomotor behaviour. Thus, we show that SoxD is required for the control of lobula plate T4 and T5 neurite guidance without affecting neuronal fate. Finally, we demonstrate that misexpression of SoxD is sufficient to alter neurite guidance in photoreceptors and mushroom body Kenyon cells.

The lobula plate is one of the less explored ganglia of the *Drosophila* visual system. Although neurogenesis has been described in some detail^8,9^, later stages of development and the mechanisms directing neuronal subtype specification are starting to be described^34^. The lobula plate has an important role in motion detection in insects, while the lobula has a role in the integration of optic stimuli and the behavioural response. Two important neuronal populations in this regard are T4 and T5 neurons, which gather information from the medulla and lobula respectively and make their synaptic outputs in the lobula plate^28,39^. Upon SoxD knockdown in all neurons (Elav-GAL4), specifically in T4/T5 neurons (IPC-GAL4 plus R42F06-Gal4) or only in T5 neurons (R42H07-GAL4), we observed defects in lobula plate morphology with increasing severity correlated with the number of affected neurons. This result suggests that SoxD may be also required for the developing of other lobula plate neurons and not only for T4/T5 neurons. In addition to RNAi-mediated knockdown experiments, we used a combination of *soxD* alleles that reduces the gene dosage. This hypomorphic condition showed a similar but milder phenotype, supporting the specificity of this phenotype to SoxD function. Furthermore, analysing SoxD knockdown phenotype in T4 or T5 neurons, we observed alterations in axon/dendrite guidance that could explain the motion perception defects of the adult animal.

Recently, Li *et al*. showed that SoxD is involved in neuronal development and degeneration. They demonstrated that SoxD is required for synaptic bouton development at the neuromuscular junction, dendritic arborisation in sensory neurons, olfactory behaviour and climbing^21^. We described a similar role for SoxD in the development of the lobula plate, but found no evidence of apoptosis upon SoxD knockdown in larval stages (data not shown), suggesting that the phenotype observed was not due to loss of lobula plate neurons but to neurite mistargeting during development. Moreover, we showed that optomotor behaviour is also affected after SoxD knockdown, complementing Li *et al*.’s behavioural observations. This evidence lends support for the role of SoxD in axon and dendrite guidance in different types of neurons of the CNS.

The mechanisms by which lobula plate neurite guidance is controlled by SoxD are unknown. Recently, it was described that Atonal promotes the differentiation of T4 and T5 neurons^33^, while Notch signalling activity discriminates between T4 and T5 neuronal fates^34^. We observed that loss of SoxD did not affect T4/T5 differentiation markers, suggesting that the function of SoxD lays downstream of the T4/T5 fate decision. Thus, we propose that SoxD controls final stages of neuronal differentiation during development.

The overgrowth of neurites observed upon SoxD knockdown in T4 and T5 neurons may result from defects in sensing an inhibitory guidance cue that restrict their growth into the medulla or the lobula. In accordance to this hypothesis, SoxD misexpression strongly affects neurite guidance in at least two different systems: photoreceptors and Kenyon cells. This supports the possible role of SoxD on sensing guidance cues. Additionally, the presence of the synaptic terminal marker in T5 dendrites after SoxD knockdown, suggests problems in neurite differentiation that could contribute to the guidance defects. Interestingly, the R42H07-GAL4 driver used to target T5 neurons was generated using an enhancer from the *soxD* locus^37^. This enhancer does not recapitulate the entire expression of *soxD*, which is also expressed in T4 neurons and other lobula plate neurons. However, SoxD knockdown was associated to an increase of the GFP fluorescence driven by R42H07-GAL4 (see levels in Fig. 6), suggesting that SoxD may negatively regulate this enhancer.

The regulation of neurite guidance may not be the only role of *Drosophila* SoxD during development. Over-expression of SoxD in embryonic neuroblasts and RNAi-mediated knockdown of SoxD in glial cells were reported to severely disrupt embryonic CNS development^22^. Surprisingly, we did not observe expression of SoxD in glial cells in the larval brain, while it remains unknown whether SoxD is expressed in embryonic glia. Furthermore, SoxD is also relevant for the function of other organs. SoxD knockdown in cardiac cells affects heart anatomy and function, while SoxD-RNAi expressed in wing discs increased the size of the longitudinal veins L2 and L3, and the marginal vein^25^.

Future work should address the signalling pathways upstream of SoxD activation and the SoxD targets that govern the morphogenesis of lobula plate neurons. Interestingly, Sox5 knockout mice show defects in axonal pathfinding of corticothalamic neurons^19^, similar to *Drosophila* T4/T5 neurons, suggesting a conserved role of SoxD proteins in neurite guidance.

A recent paper depicted the role of Sox5 in the regulation of the Collapsin Response Mediator Protein (CRMP), an intracellular protein involved in neurite guidance^42^. Interestingly the authors showed that Sox5 gain of function reduces neurite guidance through CRMP in hippocampal neurons, shedding some light to the molecular mechanism involved^42^. Future work should address the conservation of this regulation.

Finally, human Sox5 has been implicated in a number of diseases and intellectual disability in humans. Several studies report mutations and deletions in the *sox5* locus that are linked to developmental defects^43–45^. Therefore, using the fly as a model for neurite guidance may be valuable in determining the biological impact of these mutations in the onset of neurological diseases.

## Material and Methods

### Fly strains

*Drosophila melanogaster* stocks were cultured on standard medium at 25 °C. All RNAi experiments were performed at 29 °C. The following fly strains were used: *w*^*1118*^ as experimental control, Sox102F-GFP.FPTB (Bloomington #42288), UAS-Sox102F-RNAi (GD4589, VSH330016, TRiP.JF02118), UAS-shGFP-RNAi (VALIUM20-EGFP.shRNA.4), UAS-DenMark^40^, UAS-syt-eGFP, UAS-mCD8-GFP, UAS-mCD4-tdTomato, UAS-SoxD^22^, R42F06-GAL4^37^, R42H07-GAL4^37^, Elav-GAL4^C155 ^^46^, IPC-F1R3-GAL4/TM6C^28^, LexAop-mCherry, OK107-GAL4^47^, upd-GAL4^E132 ^^48^, GMR-GAL4^49^, insc-GAL4^Mz1407 ^^50^. IPC-F1R3-LexA was generated by cloning the IPC enhancer^28^ in the pLOT-attB vector.

### Immunofluorescence

Larval and Adult brains were fixed in 4% formaldehyde for 20 min and stained as previously described^51^. The following primary antibodies were used: rabbit anti-GFP 1:1000 (LifeTechnologies), mouse anti-Acj6 1:10 (DSHB), mouse anti-Elav 1:20 (9F8A9, DSHB), rat anti-Elav 1:20 (7E8A10, DSHB), mouse anti-Ey 1:20 (DSHB) mouse anti-Dac 1:40 (mAbdac2–3, DSHB), rat anti-CadN 1:20 (DN-Ex #8, DSHB), mouse anti-Chp 1:50 (24B10, DSHB), mouse anti-Fas2 1:10 (1D4, DSHB), rabbit anti-RFP 1:500 (clontech), mouse anti-Repo 1:20 (8D12, DSHB), rabbit anti-Mira 1:500, rabbit anti-pH3 1:200 (Millipore), mouse anti-Dlg 1:50 (4F3, DSHB), guinea pig anti-Dpn 1:500 (Brand lab). DNA was stained using DAPI or TOPRO-3 1:200 (Molecular Probes, Invitrogen).

Alexa Fluor conjugated secondary antibodies were diluted 1:200 (Molecular Probes, Invitrogen). Primary and secondary antibodies were incubated at 4 °C overnight. Brains were mounted on slides in Vectashield (Vector).

### *In situ* hybridisation and FISH

*soxD* probe was a kindly gift from Steve Russell. Briefly, pDmSoxD was cut with KpnI, purified and a digoxigenin-labelled probe was synthesised using T7 RNA Polymerase.

*In situ* hybridisation was performed using a standard protocol. Third instar *w*^*1118*^ larval brains were fixed in 4% Formaldehyde in 1X PBS and then washed with PBT (1X PBS, 0.1% Tween-20). Samples were permeabilised using 50 µg/mL Proteinase K. Probe was hybridised at 55 °C overnight. Then brains were blocked 30 min in 10% normal goat serum and incubated with anti-digoxigenin AP 1:2,000 (Roche) for two hrs at room temperature. Staining was performed using NBT/BCIP.

For fluorescent *in situ* hybridisation (FISH), after hybridisation brains were washed 3 times for 5 min in PBT. Endogenous peroxidase was blocked by incubating in 3% H_2_O_2_ in PBST for 30 min. Samples were washed 3 times for 5 min in PBT and 3 times for 5 min in PBTr (1X PBS, 0.3% Triton X-100). Brains were blocked with 10% normal goat serum in PBTr for 30 min. Antibodies were diluted in PBTr. Samples were incubated with primary antibodies and anti-DigPOD 1:250 (Roche) and incubated overnight at 4 °C. Samples were washed 3 times for 5 min and 3 times for 15 min in PBTr. Brains were incubated in secondary antibodies in PBTr for 2 hrs at room temperature. Samples were washed 3 times for 5 min and 3 times for 15 min in PBTr. Then they were incubated overnight at 4 °C with Tyramide Fluorophore 1:50 (Perkin Elmer) and washed 3 times for 5 min and 3 times for 15 min in PBTr. Brains were mounted on slides in Vectashield (Vector).

### Imaging and analysis

Images were acquired using a Leica SP7, a Zeiss LSM710 or an Olympus Fluoview FV1000 confocal microscopes. *In situ* hybridisation images were acquired using a Zeiss Axioplasm microscope with a Leica DFC420C camera. Images, diagrams and figures were assembled using Fiji, Adobe Photoshop CC and Illustrator CS3. Images were quantified using Fiji by measuring the area of the lobula plate and normalised by the area of the medulla in the same optic lobe plane.

### Phylogenetic analysis

Phylogenetic three was generated using Phylogeny.fr^52^ using default settings. The *Drosophila melanogaster* sequences used were: Sox102F-PA (NP_726612), Sox102F-PB (NP_001014695), Sox14-PA (NP_476894), Sox100B-PA (NP_651839), Sox15-PA (NP_523739), Sox21a-PA (NP_648694), Sox21a-PB (NP_001261827), Sox21b-PA (NP_648695), Sox21b-PC (NP_001261829), Dichaete-PA (NP_524066), SoxN-PA (NP_524735) and SoxN-PB (NP_001260269). The *Homo sapiens* sequences were: SOX-5 isoform b (NP_694534), SOX-5 isoform c (NP_821078), SOX-6 isoform 2 (NP_201583), SOX-13 (NP_005677), SOX-4 (NP_003098), SOX-11 (NP_003099), SOX-12 (NP_008874), SOX-8 (NP_055402), SOX-9 (NP_000337), SOX-10 (NP_008872), SOX-7 (NP_113627), SOX-17 (NP_071899), SOX-18 (NP_060889), SOX-1 (NP_005977), SOX-2 (NP_003097), SOX-3 (NP_005625), SOX-14 (NP_004180) and SOX-21 (NP_009015).

### Optomotor assay

For evaluating optomotor response we generated a transparent eight-choice maze based on the description from Van Swinderen *et al*.^35^ and using a 3D printing service. In a dark room, the maze was attached to a 23-inche screen, in which one cm green and black stripes were constantly moving to the right with a frequency of 3 Hz. Groups of 15–25 female flies (2–5 days old) were pushed into the start of the maze and the ending optomotor position was scored (from -4 to 4). The Optomotor Index (OI) was calculated by the following formula: OI = ∑(Number of Flies)*Optomotor Position. The average OI of 8 groups was plotted and statistical analysis was performed using GraphPad Prism 6.

## Electronic supplementary material

Supplementary Information

## Data Availability

All data generated or analysed during this study are included in this article. All reagents are available upon request.
