# Supplementary material for: The transcription factor SoxD controls neuronal guidance in the Drosophila visual system
Source: Sci Rep. 2018 Sep 6;8:13332. doi: 10.1038/s41598-018-31654-5 (PMC6127262; doi:10.1038/s41598-018-31654-5)
Supplement: Supplementary file 1 — Supplementary Information [file 41598_2018_31654_MOESM1_ESM.pdf]

SUPPLEMENTARY INFORMATION
The transcription factor SoxD controls neuronal guidance in the *Drosophila* visual system

Esteban G Contreras[1,2], Tomás Palominos[3], Alvaro Glavic[2], Andrea H Brand[4], Jimena Sierralta[1] and Carlos Oliva[3]†

SUPPLEMENTARY FIGURE LEGENDS

Supplementary Figure S1. SoxD is not expressed in glial cells (A-F) Immunofluorescence of SoxD:GFP (green) larval brains stained for GFP (green) and the pan-glial marker Repo (red). **(A-C)** Images in the antero-posterior axis and **(D-F)** sections in the medio-lateral axis. Scale bars are 20 µm.

Supplementary Figure S2. SoxD knockdown does not alter lamina neuropil morphology. (A-B') Immunostaining of **(A-A')** control and **(B-B')** shSoxD-RNAi knockdown adult optic lobes against CadN (green or gray) and Chp (red). Knockdown was performed in all LPCs during development using the upd-GAL4^{E132} driver. Scale bars are 50 µm.

Supplementary Figure S3. SoxD is necessary for the lobula plate neuropil. (A) Genome browse view of the *soxD* locus showing its four mRNA and protein isoforms in orange and green respectively. Red triangle shows the MiMIC insertion site of the *soxD*^{MI01054} in the third intron of *soxD-RB* isoform. This insertion interrupts the transcription of the *soxD-RB* isoform. (B-C') Immunostaining of **(B-B')** hemizygous control (*Df(4)O2/+*) and **(C-C')** *soxD* mutant (*Df(4)O2/soxD*^{MI01054}) adult optic lobes, against CadN (red or gray) and DNA (green). Arrows show bridges between the lobula and lobula plate neuropils. Scale bars are 20 µm. (D) Graph

showing the quantification of the lobula plate (LoP) size normalised to the size of the medulla (Me). Student's t-test was performed, n.s. means not significant difference.

Supplementary Figure S4. SoxD is required for lobula plate development. Immunostaining of insc-GAL4 **(A-D)** control and **(E-H)** shSoxD-RNAi knockdown optic lobes at **(A, E)** 48 hrs after puparium formation (APF), **(B, F)** 72 hrs APF, **(C, G)** 1 day after eclosion (DAE) and **(D, H)** 7 DAE. Brains were stained for CadN. Numbers show the frequency of the phenotype for each case. Scale bars are 20 μm.

Supplementary Figure S5. SoxD knockdown does not affect lobula plate neuronal differentiation. (A-F') Immunofluorescence of IPC-GAL4 **(A, A', C, C', E, E')** control and (B, B', D, D', F, F') shSoxD-RNAi knockdown third instar larval brains. **(A-B')** are stained for nlsGFP (driver expression in green) and Acj6 (red or gray), while **(C-D')** are stained for nlsGFP (driver expression in green) and Dac (red or gray). **(E-F')** are stained for SoxD:GFP (green or gray) and Elav (blue). Scale bars are 20 μm.

Supplementary Figure S6. SoxD controls T4 arborisation in a neuronal-autonomous manner. (A-B', D-E') Immunofluorescence of adult optic lobes of IPC-GAL4, R42F06-GAL4, UAS-CD4-tdTomato **(A-B')** control and **(D-E')** shSoxD-RNAi knockdown, against tdTomato (green or gray) and CadN (red). Arrows show T4 neurites in the medulla neuropile. Scale bars are 20 μm. **(C, F)** Schemes showing lobula plate T4 and T5 neurons in **(C)** control and **(F)** SoxD knockdown backgrounds.

Supplementary Figure S7. SoxD is not expressed in photoreceptors nor mushroom body neurons, and SoxD overexpression affects T5 neurite guidance. (A-A'') Immunofluorescence of a SoxD:GFP larval eye-antenna imaginal disc stained for GFP (green or gray), the neuronal marker Elav (photoreceptors in red or gray) and DNA (blue). Note that SoxD is only expressed

anterior to the morphogenetic furrow and not in Elav-positive photoreceptors. **(B-B'')** Immunofluorescence of a SoxD:GFP (green) larval brain stained for GFP (green or gray), the neuroblast marker Deadpan (Dpn in red) and the mushroom body lineage marker eyeless (Ey in blue and gray). Note that SoxD is not expressed in Ey-positive neurons. **(C-F')** Immunofluorescences of of adult optic lobes of R42H07-GAL4, UAS-mCD8-GFP **(C-D')** control and **(F-F')** UAS-SoxD, against GFP (green) and CadN (red). Scale bars are 20 μm.

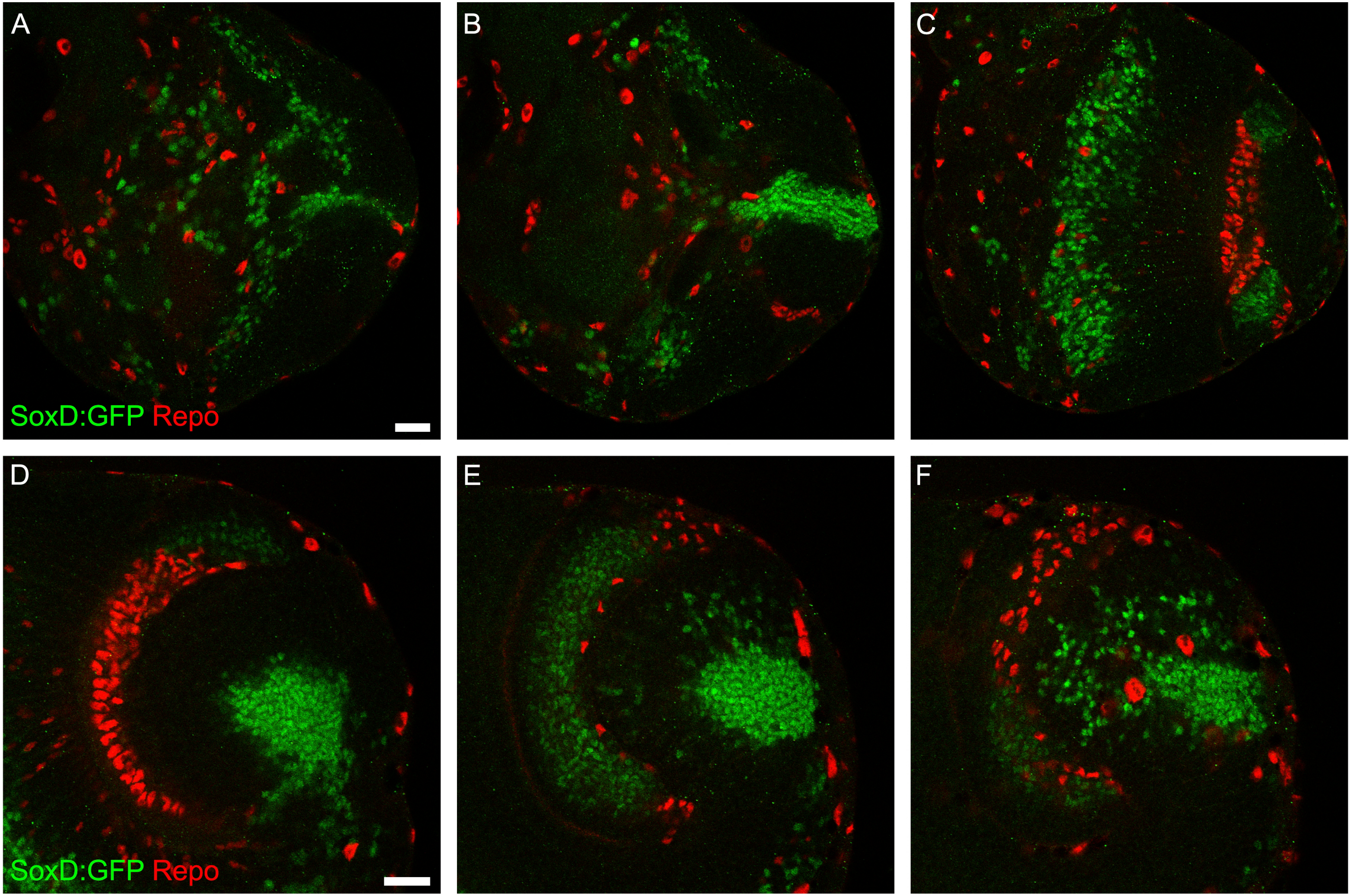

Figure S1

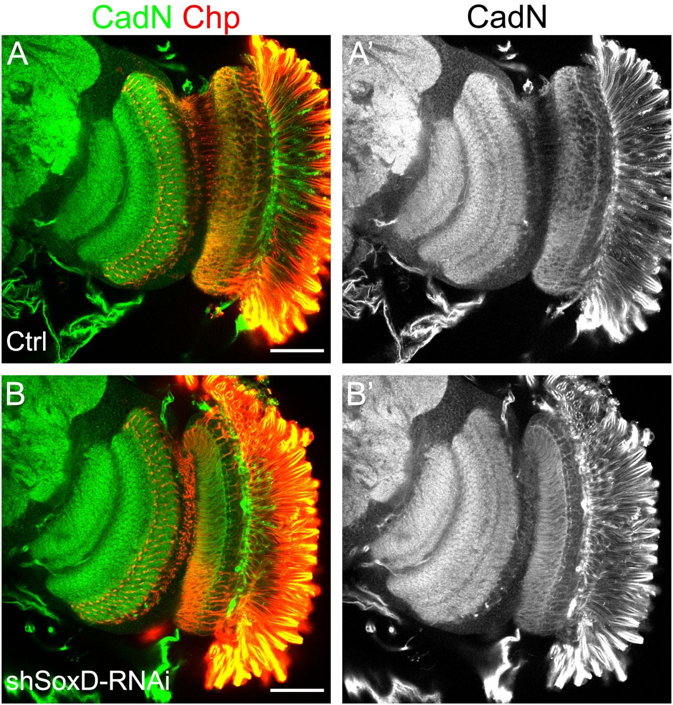

Figure S2

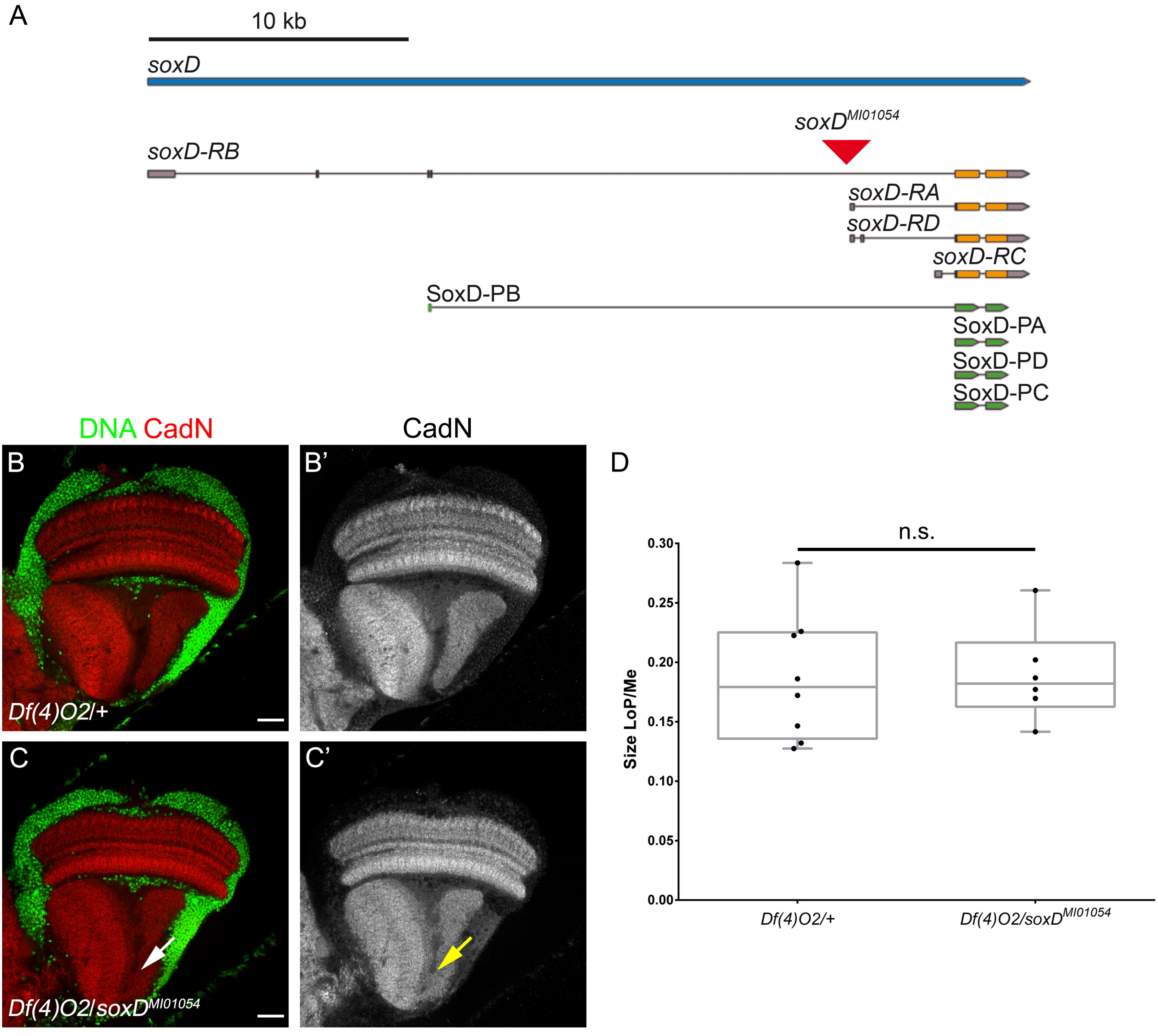
A
10 kb
soxD
soxD-RB
soxD^MI01054
soxD-RA
soxD-RD
soxD-RC
SoxD-PB
SoxD-PA
SoxD-PD
SoxD-PC
DNA CadN
CadN
B
B'
Df(4)O2/+
C
C'
Df(4)O2/soxD^MI01054
D
n.s.
Size LoP/Me
0.30
0.25
0.20
0.15
0.10
0.05
0.00
Df(4)O2/+
Df(4)O2/soxD^MI01054

Figure S3

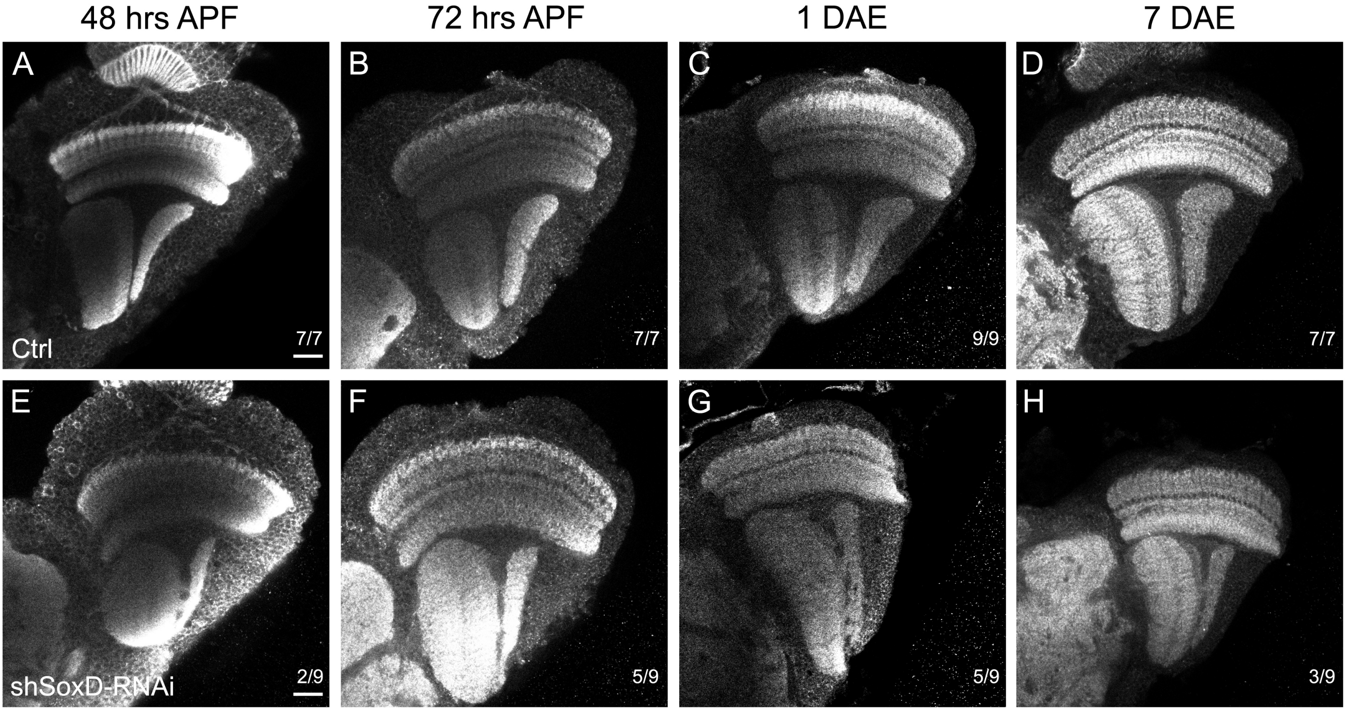

48 hrs APF
72 hrs APF
1 DAE
7 DAE
A
B
C
D
Ctrl
7/7
7/7
9/9
7/7
E
F
G
H
shSoxD-RNAi
2/9
5/9
5/9
3/9

Figure S4

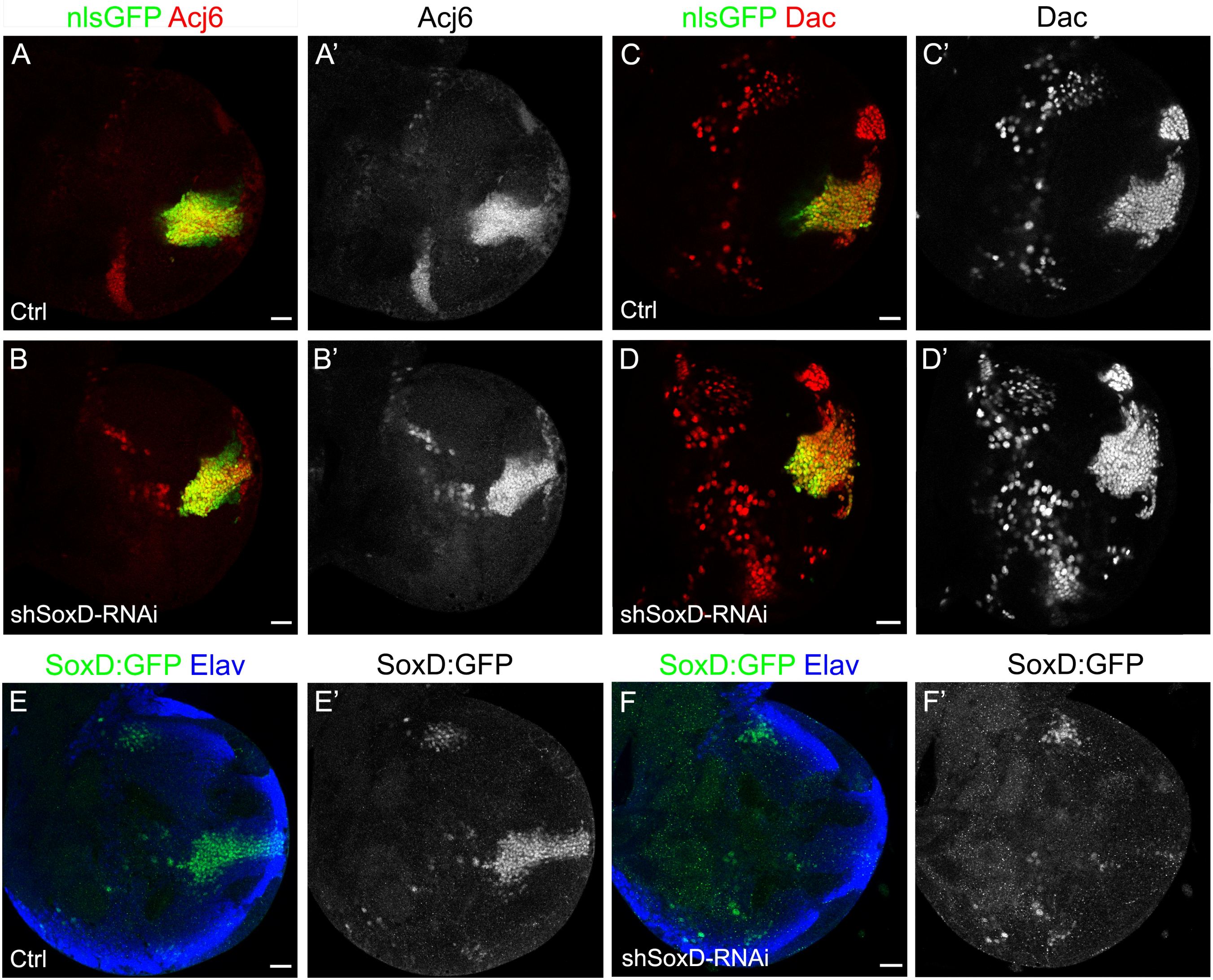
nlsGFP Acj6
Acj6
nlsGFP Dac
Dac
A
A'
C
C'
Ctrl
Ctrl
B
B'
D
D'
shSoxD-RNAi
shSoxD-RNAi
SoxD:GFP Elav
SoxD:GFP
SoxD:GFP Elav
SoxD:GFP
E
E'
F
F'
Ctrl
shSoxD-RNAi

Figure S5

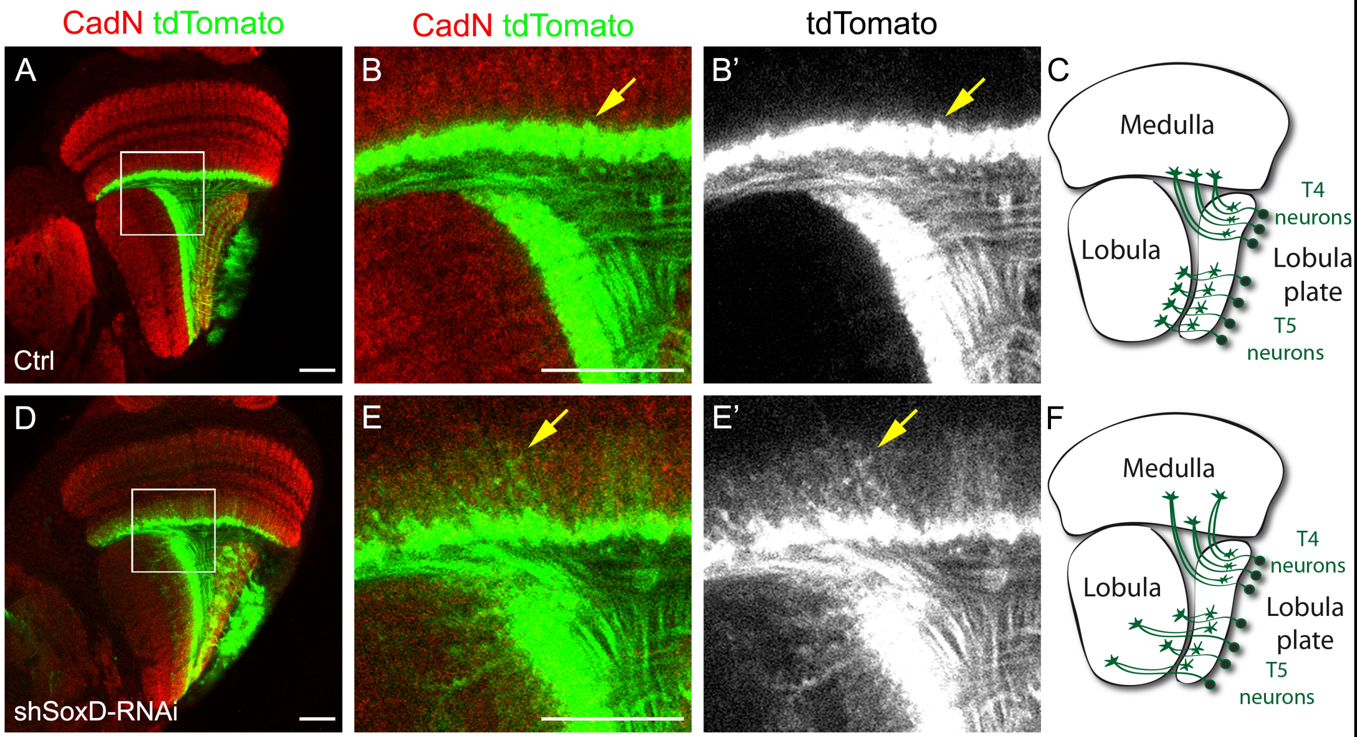
CadN tdTomato
CadN tdTomato
tdTomato
A
Ctrl
B
B'
C
Medulla
Lobula
T4
neurons
Lobula
plate
T5
neurons
D
shSoxD-RNAi
E
E'
F
Medulla
Lobula
T4
neurons
Lobula
plate
T5
neurons

Figure S6

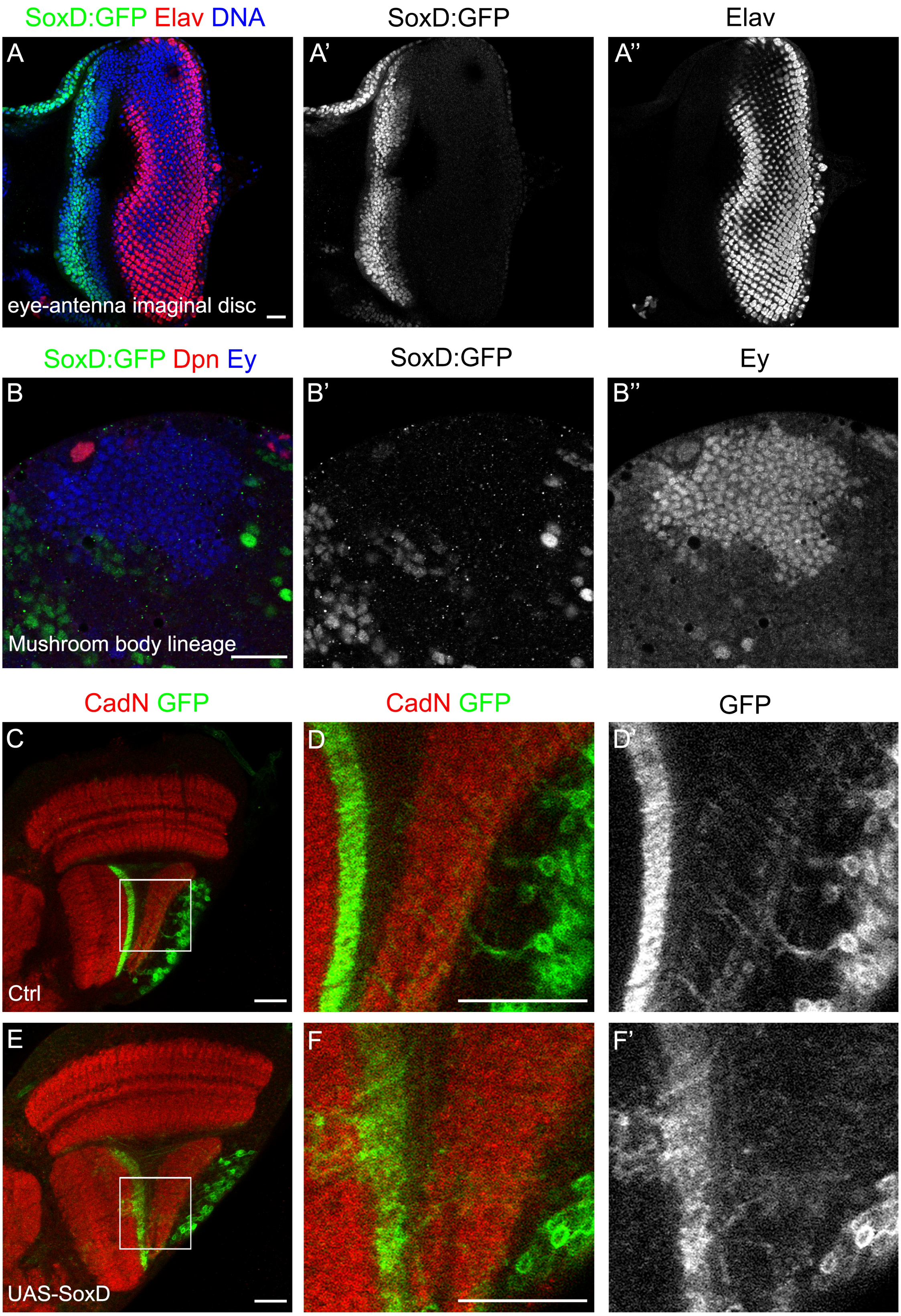
SoxD:GFP Elav DNA
SoxD:GFP
Elav
A
A'
A''
eye-antenna imaginal disc
SoxD:GFP Dpn Ey
SoxD:GFP
Ey
B
B'
B''
Mushroom body lineage
CadN GFP
CadN GFP
GFP
C
D
D'
Ctrl
E
F
F'
UAS-SoxD

Figure S7
